# Inorganic novel cubic halide perovskite Sr_3_AsI_3_: Strain-activated electronic and optical properties

**DOI:** 10.1016/j.heliyon.2023.e19271

**Published:** 2023-08-18

**Authors:** Avijit Ghosh, Md Ferdous Rahman, Md Rasidul Islam, Md Shoriful Islam, Mongi Amami, M. Khalid Hossain, Abu Bakar Md Ismail

**Affiliations:** a Advanced Energy Materials and Solar Cell Research Laboratory, Department of Electrical and Electronic Engineering, Begum Rokeya University, Rangpur 5400, Bangladesh; b Department of Electrical and Electronic Engineering, Bangamata Sheikh Fojilatunnesa Mujib Science & Technology University, Jamalpur-2012, Bangladesh; c Department of Chemistry, College of Sciences, King Khalid University, P.O. Box 9004, Abha, Saudi Arabia; d Institute of Electronics, Atomic Energy Research Establishment, Bangladesh Atomic Energy Commission, Dhaka 1349, Bangladesh; e Solar Energy Laboratory, Department of Electrical and Electronic Engineering, University of Rajshahi, Rajshahi 6205, Bangladesh

**Keywords:** Perovskite, Strain, Electronic properties, Optical properties, And optoelectronic device

## Abstract

In recent years, inorganic perovskite materials have attracted a lot of attention in the field of solar technology due to their exceptional structural, optical, and electronic properties. This study thoroughly investigated, using first-principles density-functional theory (FP-DFT), the impact of compressive and tensile strain on the structural, optical, and electrical properties of the inorganic cubic perovskite Sr_3_AsI_3_. The unstrained planar Sr_3_AsI_3_ molecule exhibits a direct bandgap of 1.265 eV value at Γ point. The bandgap of the Sr_3_AsI_3_ perovskite is lowered to 1.212 eV when the relativistic spin-orbital coupling (SOC) effect is subjected in the observations. In addition, the structure's bandgap exhibits a falling prevalence due to compressive strain and a slight rise due to tensile strain. The optical indicators such as dielectric functions, absorption coefficient, reflectivity, and electron loss function show that this component has a great ability to absorb in the visible range in accordance with band characteristics. When compressive strain is raised, it is discovered that the spikes of the dielectric constant of Sr_3_AsI_3_ move to lower photon energy (redshift), and conversely, while growing tensile strain, it exhibits increased photon energy changing behavior (blueshift). As a result, the Sr_3_AsI_3_ perovskite is regarded as being ideal for use in solar cells for the production of electricity and light management.

## 1 Introduction

The intriguing properties of organic-inorganic perovskites (OILHP), such as a fair bandgap, ubiquitous availability, outstanding optical absorption capacity, low reflectivity, and inexpensive manufacturing cost, have recently attracted a lot of experimental interest in solar technology [[1], [2], [3], [4], [5], [6]]. Although CdTe, CIGS, Sb_2_Se_3_, CMTS, and FeSi_2_ solar cell materials have made tremendous advances in the PV area [[7], [8], [9], [10], [11]], researchers have started to implement perovskites materials in the PV sector for their outstanding characteristics. In comparison to other semiconductor materials, perovskite materials are able to catch more photons while using a layer with a thickness of less than 1 μm [12]. In 2022, solar cells based on perovskite-like CsPbI_3_ can achieve a power conversion efficiency (PCE) of 17.9% with no HTL [13] and 19.06%, including the HTL layer in 2023 [14]. Solar cells connected to OILHP can display high power conversion efficiency (PCE), which, in 2022, was 25.8% [15]. This progress has been sped up by improvements to perovskite materials' properties over the past ten years. There has been a lot of interest in inorganic perovskites formed of metal halides. Unfortunately, a significant concern at the moment is OILHP's trouble with long-term durability. When used in a real setting, these perovskites' prolonged stability will be greatly affected because they are highly vulnerable to moisture, wind, sunshine, and temperature [16,17]. As a result, it has recently become very challenging to address the instability problem with these perovskites.

It is anticipated that substituting an inorganic cation for an organic cation will considerably reduce the heat instability and optical instability problems of the OILHP solar cells [18,19]. In terms of performance and stability, a few inorganic halide perovskites now outperform and stabilize OILHP [20,21]. Zhu et al. [22] discovered that inorganic halide perovskites had essentially the same band edge carrier properties. Recent research suggests that inorganic cubic perovskites are direct bandgap materials with high optical absorption, making them a strong candidate for LED, semiconducting, and solar energy technologies [[23], [24], [25]]. S. Dahbi et al. examined the electronic and optical characteristics of Lead-Free Zirconate perovskites doped with various chalcogen impurity concentrations and found that the VBM and CBM were significantly impacted [26]. Furthermore, L. Zhang et al. discovered that the perovskites solar cells with inorganic characteristics could sustainably produce large open-circuit voltages [27]. Therefore, it is envisaged that the shortcomings of OILHP would be eliminated with the effective use and production of inorganic halide perovskite materials and their photovoltaic cells. Sr_3_AsI_3_ perovskite, one of the A_3_BX_3_ type perovskite materials, has recently drawn significant interest in the field of solar technology due to its remarkable structural, optical, and electronic properties. It is a novel material as its bandgap was only calculated by the researchers in previous paper [28], and further investigations (charge density, density of state, phonon dispersion, optical properties) have been done in this paper. This perovskite has outstanding optoelectronic characteristics, including strong light absorption, lengthy carrier diffusion lengths, and effective charge transfer. It can be produced cheaply and in large quantities by synthesizing it from a solution. It is appealing for large-scale device manufacture due to its solution processability, which could result in affordable and widely available technology. Because of its highly adjustable structure, changes to its composition, crystal structure, and doping can be made to vary its characteristics. Sr_3_AsI_3_ has the potential to be used in solar cells, LEDs, photodetectors, and lasers, among other devices. Perovskite LEDs offer programmable and efficient light sources, while perovskite solar cells offer high efficiency and affordable production. High sensitivity, quick response times, and adjustable emission wavelengths are all features of perovskite-based photodetectors and lasers. However, research on Sr_3_AsI_3_ perovskite is still lacking. Consequently, it is crucial to thoroughly research Sr_3_AsI_3_ perovskite.

The bandgap significantly affects the PCE of solar cells since it is responsible for both the generation of nanoparticles and light consumption. Shockley-Queisser theory predicts that if the bandgap of perovskite solar cells' components is tuned to be between 1.2 and 1.4 eV, their PCE might reach up to 33% [29,30]. Inorganic halide perovskites are perfect materials for optoelectronic and photovoltaic systems, but they have one significant flaw: a slightly larger bandgap [31,32]. For inorganic halide perovskite solar cells to achieve the maximum PCE, the electrical bandgap controllability with various methods is, therefore, crucial [33]. A powerful technique for altering the atomic makeup and physical characteristics of perovskite materials to make them suitable for solar applications is strain technology. The latest research on strain-induced material characteristics has revealed a clear interlinkage between the element used and its structural characteristics [[34], [35], [36], [37], [38], [39], [40]]. For organic-inorganic tri-iodide perovskite materials, it has been observed that applying a small amount of pressure (below 0.3 GPa) causes a beneficial bandgap reduction and increases the carrier lifetime by 70% to 100% [41]. In a detailed investigation of CsPbI_3_ under strain, Jing et al. [42] found that by varying the strain insertion in the range of 5% to 5%, the bandgap of CsPbI_3_ can be adjusted between 1.03 and 2.14 eV. Mohamed Abdelilah Fadla et al. [43] investigated how the iodine partial replacement greatly increases the material stability in CsPbI_3-x_Br_x_ perovskites. According to A. K. Hossain et al. [44], the cubic CsSnCl_3_, a perovskite of inorganic characteristics, can convert a semiconductor into a metal substance with excellent optical and electronic properties under compressive strain. By putting stress on CH_3_NH_3_GeI_3_, one can increase the material's potential for use in photovoltaics by achieving a greater band gap energy, increased mobility of carrier, and enhanced the coefficients of absorption [45]. According to D. Liu et al. [46], it is possible to successfully modify some parameters of CsGeI_3_ material, such as bandgap, and dielectric coefficient by applying compressive and tensile strain. The researchers demonstrated how the impacts of strain reduce the band gap energy in the perovskite compound CsPbX_3_ [24]. The amount of applied compressive and tensile strain greatly influences the structural, electronic, and optical characteristics of Sr_3_AsI_3_ perovskite. Higher cations have more nucleons than smaller ones due to their higher atomic sizes. The ensuing variation in atomic size leads to a shift in the electronic band structure. Broadly speaking, the SOC effect can change a variety of materials' electrical characteristics [47,48]. According to a paper, the band structure of halide perovskites could result in rending and a bandgap fall of about ∼1 eV when taking the SOC effect into account [49]. As a result, it is imperative to carry out a thorough and methodical investigation of the SOC effect, and biaxial strain fluctuation of APbBr_3_ perovskite. Therefore, a comprehensive study about the impact of strain and SOC effect on Sr_3_AsI_3_ perovskite is very significant.

In this research, the density functional theory (DFT) approach is used to extensively analyze the strain-induced optical and electronic properties of Sr_3_AsI_3_. We thoroughly investigated Sr_3_AsI_3_'s band structure and bandgap customization process. We have mainly concentrated on how the SOC affects the electronic characteristics of Sr_3_AsI_3_ perovskite. To describe the electronic characteristics of Sr_3_AsI_3_, the impact of outside strain just on the modification of both the energy gap and structural characteristics was explored. When attempting to understand the shifting characteristics of blue and red of the maxima of dielectric at various strain applications, the absorption spectrum properties of Sr_3_AsI_3_ were taken into consideration. One can design the Sr_3_AsI_3_ material to be suitable for optoelectronic and photovoltaic technologies by modifying its optoelectronic properties.

## 2 Details of computational method

The norm-conserving (NC) pseudopotential [[50], [51], [52]] and the Perdew-Burke-Ernzerhof (PBE) [53] exchange-correlation mechanism were used to transform the Sr_3_AsI_3_ perovskite structure to the FP-DFT. It was predicted that the Quantum Espresso simulation program would produce the DFT [[54], [55], [56], [57]]. The required initial settings were present in the input data, including the Brillouin zone grid, crystal forms, lattice parameters, and kinetic cut-off energy. The structure's kinetic energy cut-off and charge density cut-off were changed to 30 Rydberg (Ry) and 220 Rydberg (Ry), respectively, to improve efficiency and performance. When using the vc-relax calculation to optimize the lattice, the dimension of the k-point (k_x_,k_y_,k_z_) was set to (6 × 6 × 6). The highest set of force tolerance at 0.01 eV/Å and a convergence threshold value of 106 a.u. were used to derive the self-consistency equation [58]. In structural and ionic adjustment relaxation investigations, the force convergence threshold of about 10^−3^ a.u. was considered. Although there are certain ways to lessen this error, we have not used corrected PBE for metals in the most recent studies [59,60]. By varying the lattice parameter a_strained,_ the biaxial compressive and tensile strain was reproduced. We used equation (1) for calculating strain [61],

(1) $$\varepsilon =\frac{a_{strained}-a_{relaxed}}{a_{relaxed}}\times 100\%$$

The unstrained lattice constant is referred to as a_relaxed_ in equation (1). The range of ε , expressed in increments of 1%, ranges from −4% to +4% and correspondingly, compressive and tensile strains are indicated by negative and positive strain values. Dynamically stabilized, the perovskite framework's optical properties were examined by computing their intricate, photon energy-sensitive dielectric functions. The optical properties were computed using the theory of time-dependent first-order perturbation provided by the QE program [62]. Consequently, the complex dielectric component was studied to ascertain the photon's energy spectrum (eV) at which it exhibits absorption peaks. The complex dielectric function, $\varepsilon \left(\omega \right)=\varepsilon_{1}\left(\omega \right)+i\varepsilon_{2}\left(\omega \right)$, is recognized as the essential relationship for measuring optical absorption coefficients. In order to accurately depict the band structures caused by the large arsenic element, the SOC effect was also taken into account in the DFT investigation.

## 3 Result and discussion

### 3.1 Structural properties

**Fig. 1:**
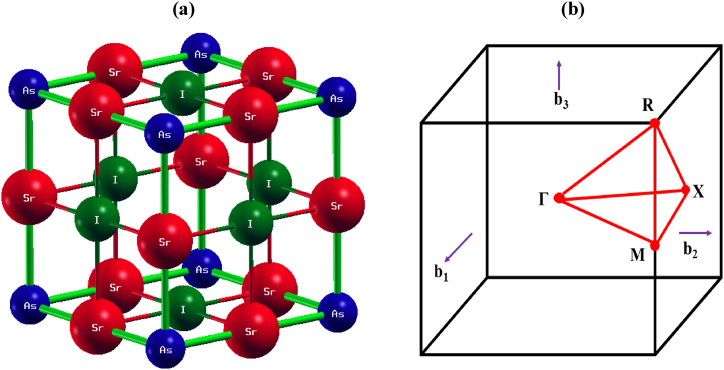
(a) The most ideal structure of the inorganic perovskite Sr_3_AsI_3_. (b) The k-path of the first Brillouin zone to identify the electronic band structure.

The periodic pattern of Sr_3_AsI_3_ has been discovered to be the Pm3 m cubic foundation [63]. Seven atoms comprise the structure's unit cell. This substance consists of a face-centered cubic lattice of Sr and As atoms and an octahedral gap filled by I atoms. Both Sr–As' bonds are 2.84 Å long. Sr-l bonds typically have a length of 2.84 Å. As^3−^ is connected to six equivalent Sr^2+^ atoms to form corner-sharing AsSr_6_ octahedra. There is no angle between the octahedra that share a corner. The fractional coordinates of As, I, and Sr using the 1a, 3c, and 3d Wyckoff sites, respectively, are (0,0,0), (0.5,0.5,0), and (0,0.5,0), as shown in Fig. 1(a). The k-path of the first Brillouin zone (BZ) is shown in Fig. 1(b). It is required to compute structural properties before computing the various properties of Sr_3_AsI_3_ perovskite. The structural features were derived using PBE, including the lattice constant value a(Å) which is illustrated in Table 1. By balancing the overall quantity of energy against the lattice parameter, the lattice constant for Sr_3_AsI_3_ that is most environmentally friendly has been found. The combination Sr_3_AsI_3_ is estimated to have a lattice constant of a = 6.58 Å.

**Table 1:** The lattice constant and energy bandgap of Sr_3_AsI_3_ were calculated using experimental data and earlier DFT studies.

Structure	Lattice constant (Å)		Bandgap Energy (eV)	
	This work	Previous work	This work	Previous work
Sr_3_AsI_3_	6.58	6.58 [28]	1.265 (PBE) 1.94 (HSE)	1.94 (HSE) [28]

Additionally, the conjunctive and production energy of a certain structure can be employed as effective tools for verifying the robustness of such a structure [[64], [65], [66]]. It is possible to calculate these energies using equation (2):

(2) $$E_{Formation}=E_{{Sr}_{3}{AsI}_{3}}-E_{SrI}-E_{AsI_{2}}$$

By using equation (2), we have found that the negative formation energy of Sr_3_AsI_3_ exists. This perovskite structure, Sr_3_AsI_3_, displays negative conjunctive and formation energy, demonstrating its stability.

### 3.2 Phonon dispersion and density of phonon states

**Fig. 2:**
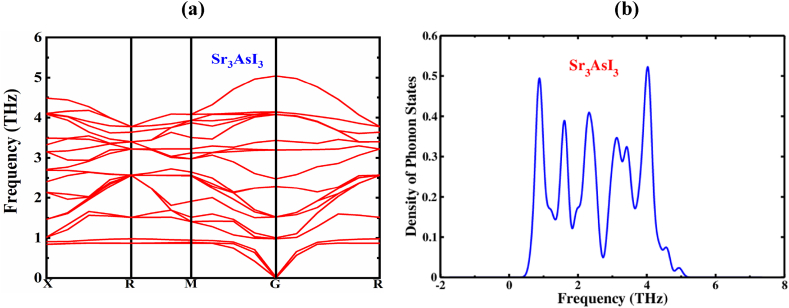
(a) The phonon dispersion spectrum and (b) Density of phonon states of Sr_3_AsI_3_ perovskite.

Phonon dispersion refers to the relationship between the energy and momentum of phonons in a crystal lattice. Phonons are quantized lattice vibrations, and their dispersion provides crucial information about the vibrational properties and thermal behavior of materials. The lattice volume and atomic positions were fully relaxed before the phonon computation was carried out [67,68]. Along with the high-symmetry points of X-R-M-G-R in the first Brillouin zone, the phonon bands were calculated for the Sr_3_AsI_3_ perovskite. Fig. 2(a)'s dispersion map can be examined to see that the dynamical matrix does not contain any negative frequencies for the approximation, demonstrating the perovskite's dynamic stability.

The number of phonon modes per unit energy interval at a specific frequency is represented by the phonon density of states. It displays the relative strengths of the different phonon modes across a variety of energies. Peaks or features in the phonon density of state can be connected to certain atomic or lattice vibrations in the material, and they correlate to particular vibrational modes [69]. Fig. 2(b) shows the phonon density of state of Sr_3_AsI_3_ for the range of −2 to 8 THz. Most of the phonons are occupied in the region 0 to 5 THz. The maximum peak of the density of phonon states is around 5.2 eV. It also has an impact on thermal conductivity since it controls the phonon modes that can be used for scattering and energy transmission.

### 3.3 Charge density

The investigation of electronic charge density is a crucial component of the evaluation of a component's electronic properties. Concerning the overall concentration of charge in a structure's unit cell, this feature generates a charge density structural map of the valence electrons. The type of chemical bonding within a molecule is investigated by looking at the total electronic charge density map.

**Fig. 3:**
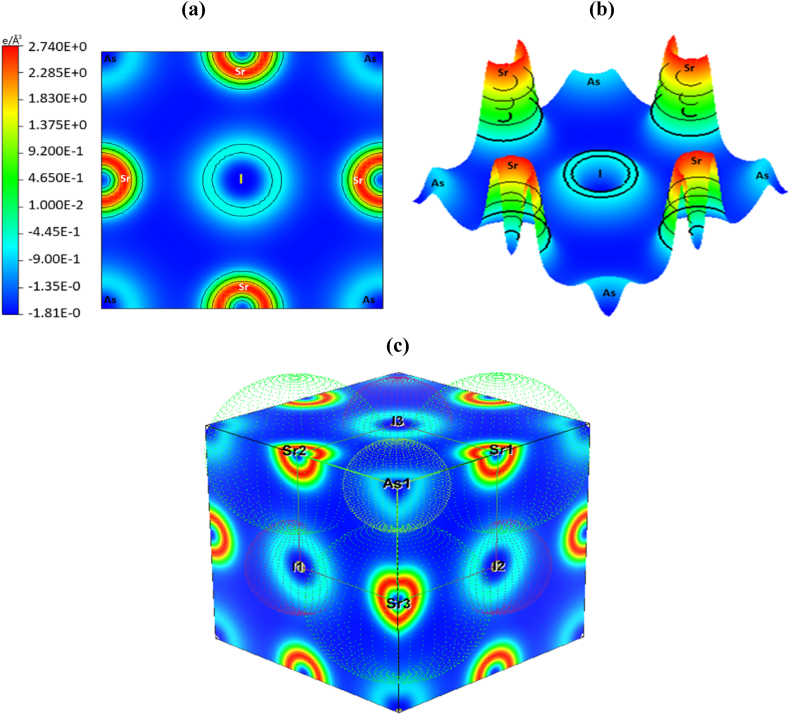
The charge density of Sr_3_AsI_3_ structure (a) 2D view, (b) Bird's eye view, (c) 3D view.

The charge density curve, which typically consists of structural atoms, illustrates how orbital electrons' ability to accumulate charges influences the electrical characteristics of atoms. The charge contributions from the distinct elements' electronic DOS spectrum are then introduced and corroborated by the differentiated color density map. Fig. 3(a) and (b), and 3(c) show the mapping image of the electron density in 2D view, bird's eye view, and 3D view. High and low intensities are represented by the colors red and blue, respectively. It is clear that on all planes, the Sr element is the area where charge builds up the fastest, whereas the As atom is where it depletes. In other words, a covalent bond is suggested by the crossing of the outer electrons between these two parts [70,71]. This charge distribution method provides strong evidence for the Sr–As atoms' covalent bonding property. The researchers claim that the charge density surrounding the atoms is nearly spherical, which indicates the presence of ionic bonding similar to that found in previously reported perovskites [72,73]. Here, Ionic bonds are indicated by the bonding of the Sr and I atoms. On the other side, As–I bonds are antibonding features because they have negative population values.

### 3.4 Electronic properties

**Fig. 4:**
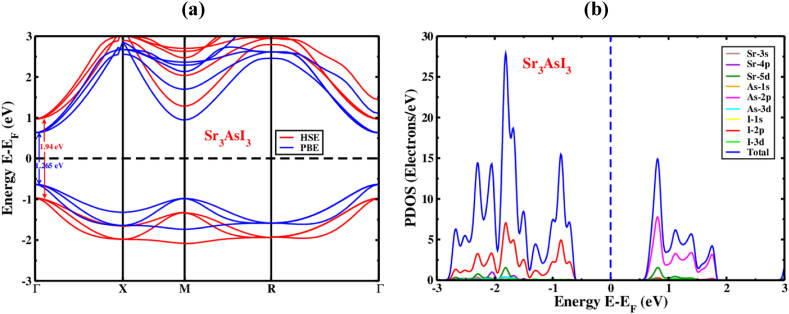
The electronic (a) band structure and (b) refined structure of PDOS for inorganic Sr_3_AsI_3_ perovskite.

Band structure, charge density, and density of states (DOS) are the main factors that affect a substance's electronic properties [74]. The high equipoise directions and the electronic band characteristics for Sr_3_AsI_3_ perovskite structures have been calculated. The electronic band configurations of the unstrained Sr_3_AsI_3_ framework are illustrated in Fig. 4(a). In order to quickly determine the size of the bandgap, the fermi levels have undergone a zero correction. Along with the k-axis, the cubic structure's Γ-X-M-R-Γ is considered. Fig. 4(a) displays the conduction band minimum (CBM) and valence band maximum (VBM), both of which are situated close to the Γ(Gamma) point. The Sr_3_AsI_3_ perovskite is projected to have direct bandgap structures with values of roughly 1.265 eV/1.94 eV based on calculations utilizing the PBE/HSE function for Sr_3_AsI_3_. This result is generally compatible with the values that were previously published [75,76]. A very common problem with the GGA method is that the bandgap value was plainly underestimated when the bandgap was evaluated. Additionally, bandgap undervaluation in the (LDA)+U and LDA techniques of approximating local density was found [77]. To prevent this type of bandgap computing, several specialists have offered a range of solutions, including the GW methodology hybrid functional [78,79].

In general, the partial density of states (PDOS) illustrates how certain atoms and their varied forms influence the bandgap energy of the Sr_3_AsI_3_ structures. Fig. 4(b) shows the PDOS dispersion in Sr_3_AsI_3_ for the range of −3 to 3 eV. In Sr_3_AsI_3_, the forms of Sr and As hybridized with I are shown to grow across the entire energy spectrum while maintaining the bandgap. It reveals that covalent bonding is the most fundamental type of bonding between Sr–I and As–I. Sr_3_AsI_3_ also involved the transfer of electron charge from Sr and As to I (Fig. 4(b)), which may be accounted for by the extreme contrast between the atomic states. The contribution of Sr ^+^ atoms is almost nonexistent in the region close to the Fermi level. Our cubic phase study analyzes the density of states close to the valence band of Sr_3_AsI_3_ and finds that the I-2p orbital dominates the analysis, whilst the As-2p orbital and a modest contribution from the Sr-3s orbitals both play a substantial role in the conduction band.

### 3.5 The SOC effect on electronic structure

**Fig. 5:**
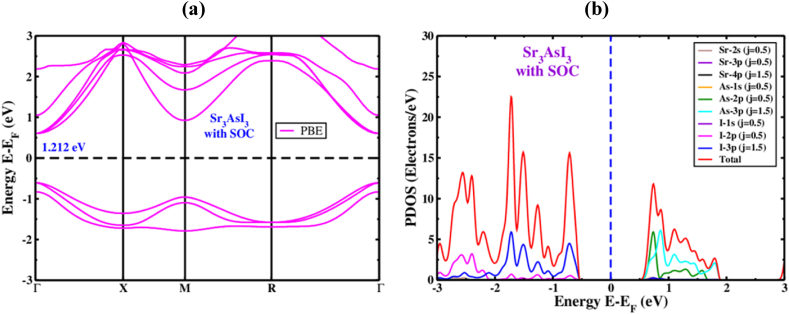
The electronic (a) band structure and (b) refined structure of PDOS for inorganic Sr_3_AsI_3_ perovskite with the function of PBE in the presence of SOC effect.

The SOC effect has been taken into account in the computation to accurately predict the band structure because of the presence of large arsenic ions in the perovskite structure. Fig. 5(a) shows that the SOC effect considerably impacts both the conduction band section and the valence band sector when the levels of CBM and VBM are modified. The CBM altered in the descending movement toward the Fermi level, while the VBM fundamentally changed in the ascending movement. Furthermore, the equally spaced point in the framework is the only spot where the criterion of spin-degeneracy is false since inorganic perovskite crystals lack inversion symmetry, which results in a band separation [47,48]. Overall angular momentum, which would be created by ls coupling, can be used to understand the band separation. Here, the angular momentum of spin is s, and the angular momentum of orbital is l. The J has a value between |l - s| and |l + s|. According to the theory of molecular orbital, the values of l and j in s orbitals are 0 and 1/2, in p orbitals are 1 and (1/2, 3/2), as well as in d orbitals are 2 and (3/2, 5/2). S is equal to +1/2 and −1/2 for every orbital. While taking into account the SOC impact, we noticed the bandgap narrowing. The Sr_3_AsI_3_ compound possesses an energy band gap of 1.212 eV rendering the conduction and valance band towards the Fermi level (Fig. 5(a)). The computed bandgap values for the Sr_3_AsI_3_ perovskite both with and without SOC are displayed in Table 2.

**Table 2:** A cubic Sr_3_AsI_3_ perovskite's predicted bandgap under various tensile as well as compressive strains with and without SOC effect.

% of strain value	Compressive strain's bandgap value (eV)		Tensile strain's bandgap value (eV)	
	Absence of SOC	Presence of SOC	Absence of SOC	Presence of SOC
0	1.265	1.212	1.265	1.212
1	1.206	1.161	1.315	1.265
2	1.139	1.095	1.361	1.318
3	1.079	1.027	1.417	1.356
4	1.004	0.950	1.447	1.408

The matching PDOS under the influence of SOC was examined to obtain a more in-depth understanding of the band structure of cubic Sr_3_AsI_3_. The PDOS, as shown in Fig. 5(b), confirms that in the presence of the SOC effect, the influence of the Sr atom does not adhere to any set of rules. Despite the SOC effect separating I-2p and I-3p into p (j = 1/2) and p (j = 3/2), the band edge is not separated at the high symmetry positions. As seen in Fig. 4(b), the I-2p (j = 1/2) as well as I-3p (j = 3/2) atoms donate the majority of the energy in the range of −3 to −0.6 eV to the VB. On the other hand, As-2p (j = 1/2) as well as As-3p (j = 3/2) make up the energy in the CB side between 0.6 and 1.9 eV.

### 3.6 Strain-induced electronic properties

**Fig. 6:**
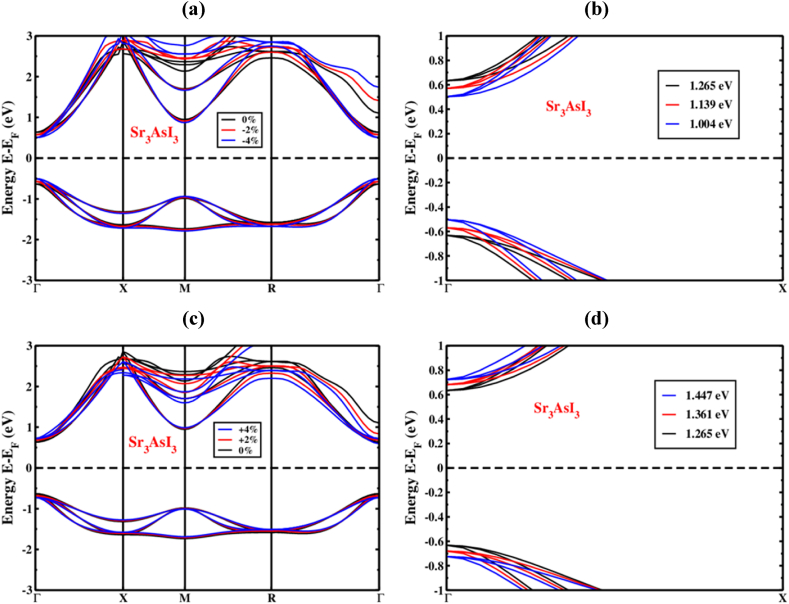
The Sr_3_AsI_3_ perovskite electronic band structure under (a) compressive and (b) zoom view of compressive strains, (c) tensile and (d) zoom view of tensile strains without SOC effect.

**Fig. 7:**
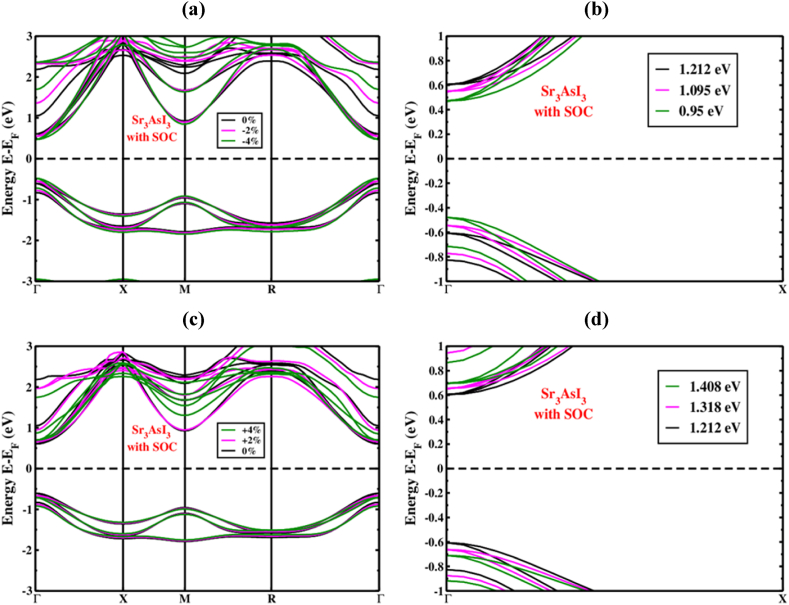
The Sr_3_AsI_3_ perovskite's electronic band structure under (a) compressive and (b) zoom view of compressive strains, (c) tensile and (d) zoom view of tensile strains with SOC effect.

We looked at how applied strain (%) influenced the Sr_3_AsI_3_ structure under tensile and compressive strains with and without applying the SOC effect. A 1% spacing had been used to vary the compressive-to-tensile strain from −4% to +4% [58]. The VBM and CBM of the Sr_3_AsI_3_ (Fig. 6(a)) frameworks migrated toward the level of Fermi energy, whereas the CBM as well as VBM remained at the Γ (Gamma)-point under compressive strain (−4% to 0%). Fig. 7(a) depicts the band configurations under compressive strain while taking into account the SOC effect of the Sr_3_AsI_3_ formation.

Because of the orbital clashing, the bond length among the Sr, As, and I was shortened when the SOC effect was included or excluded for the compressive strain. Thus, whether or not the SOC effect is included, the straight bandgap is established at the Γ (Gamma)-point. We observe that higher compressive strain is shown to minimize the bandgap with and without counting the SOC effect. On the other hand, the effect of applied tensile strain (0% to +4%) on the electronic band structure is also depicted in Fig. 6(c) for Sr_3_AsI_3_ perovskite. Fig. 7(c) depicts the band configurations under tensile strain while taking into account the SOC effect of the Sr_3_AsI_3_ formation. In response to applied tensile strain, the bandgap has increased, as evidenced by the CBM as well as VBM's deviation from the level of Fermi energy. Fig. 6(b), (d), and 7(b), 7(d) show the zoom view of Γ (Gamma)-point when the SOC effect was included or excluded both for the compressive and tensile strain.

A decrease in force between the atoms of Sr, As, and I was caused by the bond's lengthening due to tensile strain, which also caused an increase in atomic distance. Thus, whether or not the SOC effect is included, the straight bandgap is also established at the Γ (Gamma)-point. We observe that higher tensile strain is shown to maximize the bandgap, whether or not taking the SOC effect.

**Fig. 8:**
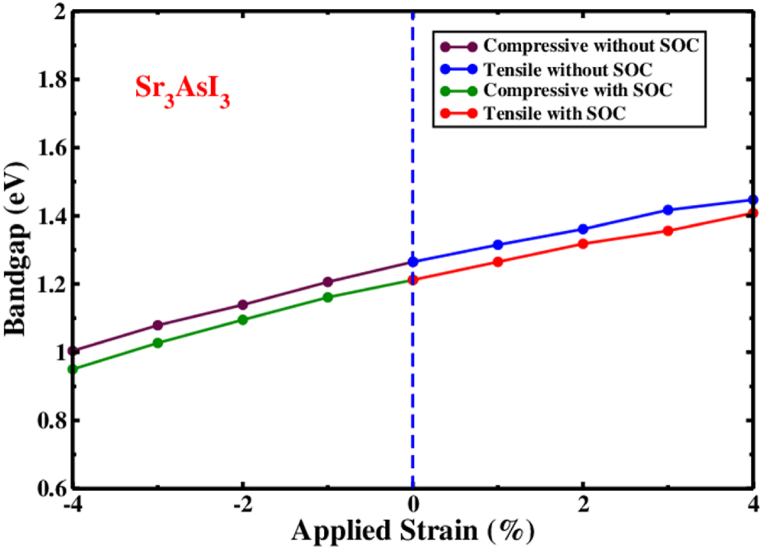
The Sr_3_AsI_3_ structure's energy bandgap in relation to the strain being applied whether or not introducing the SOC effect.

As a result of compressive and tensile strain on the Sr_3_AsI_3_ structure, the electronic band structure is a direct bandgap. Fig. 8 and Table 2 provide a summary of the bandgap modifications of the Sr_3_AsI_3_ structure under compressive and tensile strain. The bandgaps of Sr_3_AsI_3_ shifted from 1.004 to 1.447 eV (without SOC) and 0.950 to 1.408 eV (with SOC) when −4% to +4% of applied strain was used. It is interesting to observe that Sr_3_AsI_3_ maintains its direct bandgap feature across the entire range of applied strain. According to the Shockley-Queisser hypothesis, it states that this structure can be employed to improve the capacity of a solar cell [29].

**Fig. 9:**
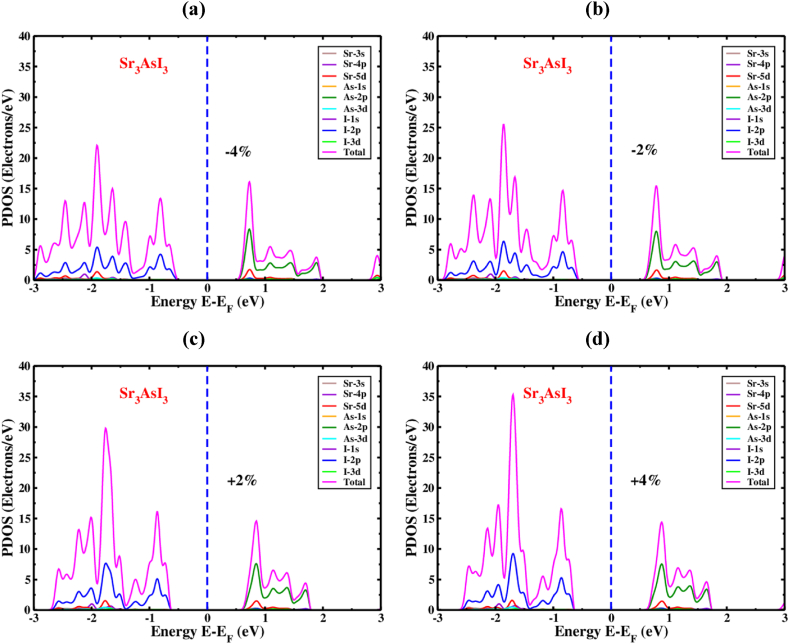
The Sr_3_AsI_3_ perovskite's PDOS under various compressive as well as tensile strain (a) −4%, (b) −2%, (c) +2%, and (d) +4%.

Moreover, as strain is applied, the orbital distributions of the particles (Sr, As, and I) fluctuate. PDOS, the partial density of states of Sr_3_AsI_3_ under induced compressive and tensile strain, is displayed in (Fig. 9a and b) and (Fig. 9c and d) without considering the SOC effect. The 2p orbital of the I atom exhibits activity towards the side of VBM, a little lower than the level of Fermi energy when the applied strain shifts from −4% to +4%. As atoms' 2p orbital controls the majority of the overall DOS in the conduction band section. Although the DOS's stable placement and configuration represented by As's 2p orbital can't affect considerably by the variation of induced strain on the section of CBM, the quantity of total DOS performance increments due to varying strain from −4% to +4%. Fig. 9 remarks that the total DOS for the Sr_3_AsI_3_ framework is 23 electrons/eV at −4% strain as well as 36 electrons/eV at +4%. While visible throughout the whole energy spectrum, the hybridized As–I and Sr–I orbitals are not visible in the bandgap zone.

**Fig. 10:**
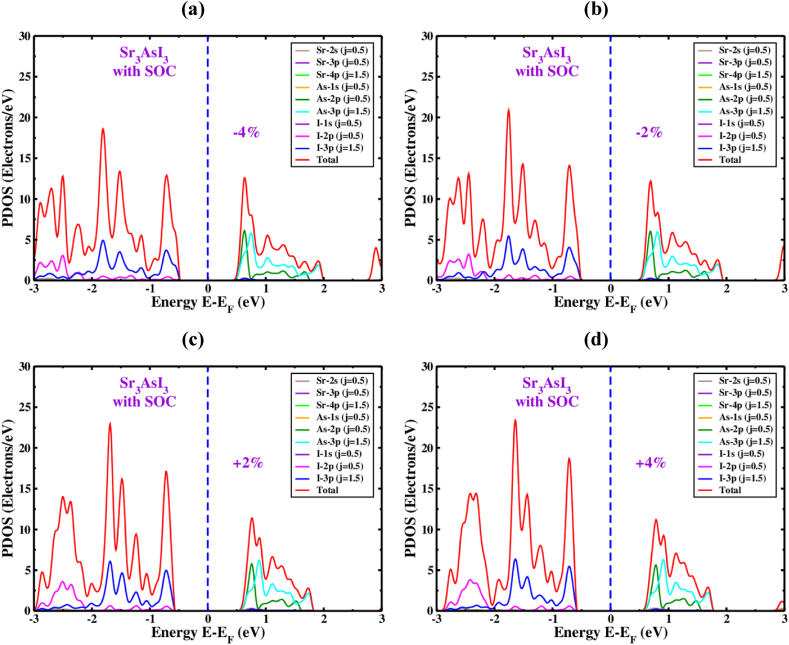
The Sr_3_AsI_3_ perovskite's PDOS under various compressive as well as tensile strain (a) −4%, (b) −2%, (c) +2% and (d) +4% with SOC effect.

PDOS, the partial density of states of Sr_3_AsI_3_ under induced compressive and tensile strain is displayed in (Fig. 10a and b) and (Fig. 10c and d) considering the SOC effect. When the applied strain changes from −4% to +4% with the SOC effect, the I atom's 2p and 3p orbitals display activity toward the side of VBM, just below the level of Fermi energy. Hence the majority of the total DOS in the section of CBM is controlled by the 2p and 3p orbitals of As atom. The quantity of overall performance of DOS rises when the applied strain varies from −4% to +4%, considering the SOC effect. According to Fig. 10, the total DOS for the Sr3AsI3 structure is 18 electrons/eV at strains of −4% and 23 electrons/eV at a strain of +4%, considering the SOC effect.

**Fig. 11:**
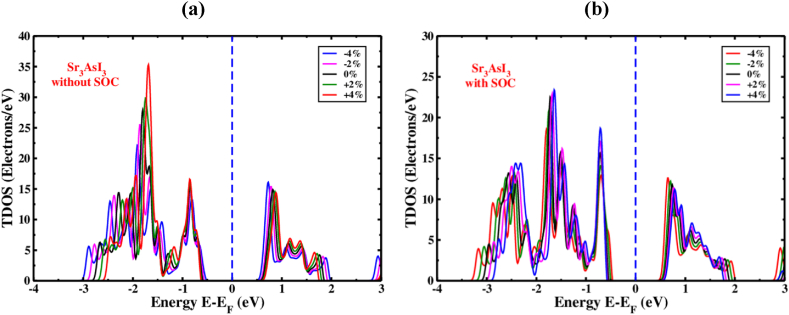
The amount of TDOS of Sr_3_AsI_3_ near the Fermi level under different strains (a) without and (b) with SOC effect.

TDOS refers to the total density of states. TDOS of Sr_3_AsI_3_ introducing various compressive as well as tensile strains without (Fig. 11(a)) and with (Fig. 11(b)) considering SOC effect is represented in Fig. 11. We can comprehend more the behavior of the Sr_3_AsI_3_'s structure of the electronic band with the help of TDOS. In unstrained Sr_3_AsI_3_, the orbitals of the I atom actually contributes to the TDOS of the valance band below E_F_, with a negligible impact from the Sr-5d and As-3d orbitals with the presence and absence of SOC effect. The orbitals of As atom basically contributes to the TDOS of the conduction band above E_F_, with a negligible impact from the Sr-5d and I-2p orbitals with the presence and absence of SOC effect. A bandgap and the material's congenital semiconducting properties are both highlighted by the disappearance of a TDOS line in the area of the level of Fermi energy level. Nayak et al.‘s experiment on MoS_2_ demonstrated that the configuration of the band and amount of bandgap of the material may change significantly when the pressure changes while staying unaffected by the several types of functional exchange-correlation used [80,81]. The TDOS line shifts in the direction of the fermi level at compressive strains (0% to −4%) with the presence or absence of the SOC effect, which increases the conductivity of Sr_3_AsI_3_ materials. On the other hand, the TDOS line deviates from the fermi level under tensile strains (0% to +4%) with and without considering the SOC effect, which decreases the conductivity of Sr_3_AsI_3_ materials. A thorough investigation of the band structure and density of the state of Sr_3_AsI_3_ predicts such a bandgap transition.

### 3.7 Optical properties

A study of the complicated dielectric functions, electron loss function, absorption coefficient, and reflectivity are included in an optical quality assessment to determine whether the materials under consideration are acceptable for optoelectronics and solar cell technologies. By modifying the lattice parameter, a thermodynamic method known as biaxial strain can improve a substance's optical performance [61]. The stretch-ability of various optical properties of Sr_3_AsI_3_ was explored and demonstrated in this study with compressive (0% to −4%) as well as tensile (0% to +4%) strains. The symbol ε(ω) represents the dielectric function. It is calculated by summing two portions, one of which is real and marked by ε_1_(ω) and the other imaginary and denoted by ε_2_(ω)

(3) $$\varepsilon \left(\omega \right)=\varepsilon_{1}\left(\omega \right)+i\varepsilon_{2}\left(\omega \right)$$

**Fig. 12:**
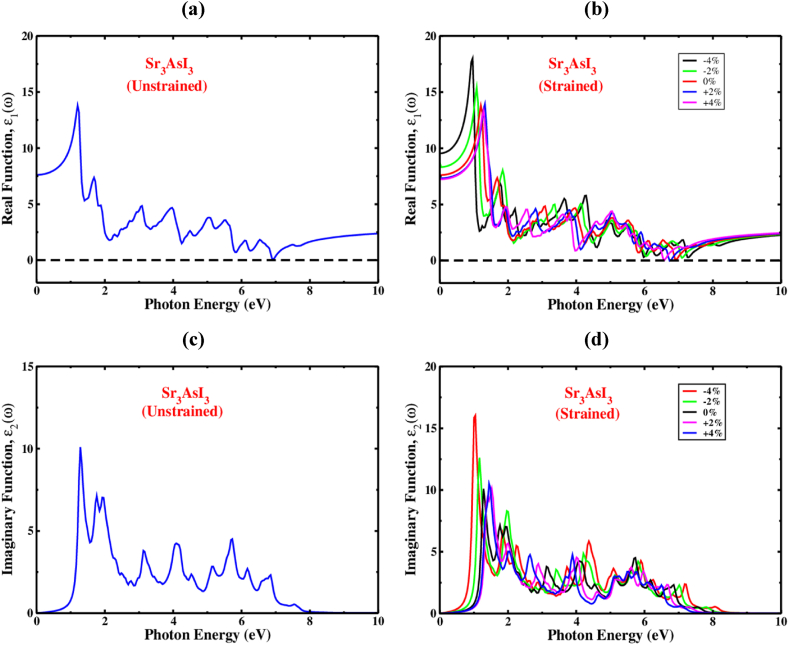
The real and imaginary portion of dielectric function with a photon energy of (a,c) without strained (b,d) with strained of Sr_3_AsI_3_.

To derive the actual dielectric function, the Kramers-Kronig transformation [82] is employed, and the elements of the momentum matrix [83] are used to calculate the imaginary portion. Fig. 12(a) and (b) show the unstrained and strain-induced real portions of the dielectric permittivity of Sr_3_AsI_3_ at photon energy up to 10 eV. To understand the effects of polarization and dispersion, the real component of the dielectric constant can be used.

The finest fundamental factor in the real state section of the dielectric function, denoted by the abbreviation ε_1_ (0), which corresponds to the electronic component, is the greatest frequency of zero. The computed value of ε_1_ (0) for cubic Sr_3_AsI_3_ is 7.55 (Fig. 12(a)). The quantity of ε_1_(ω), which increased when the material was exposed to optical excitation between ε_1_ (0) and the maximum amount of ε_1_(ω), then quickly reduced, demonstrating the material's significant capacity for light absorption in this spectral region. Additionally, the unstrained Sr_3_AsI_3_ exhibits positive ε_1_(ω) values, demonstrating that it is highly reflective and semiconducting. By altering the biaxial strain, the maxima of the dielectric constant of real sections of the Sr_3_AsI_3_ perovskite have been transformed. Generally speaking, low band gap materials have more significant dielectric constant peaks than greater bandgap components. As a result, the Sr_3_AsI_3_ structure has a higher dielectric constant peak, and the transition occurs where the photon energy is narrow (redshift) since the growing compressive strain causes the bandgap to reduce. With growing tensile strain, it demonstrates a reduced dielectric constant peak and migrates where the photon energy is high (blueshift) for the Sr_3_AsI_3_ framework.

Fig. 12(c) and (d) show, respectively, the properties of the imaginary component, ε_2_(ω), of the dielectric function under unstrained and strained conditions. The imaginary part of the dielectric function is crucial for understanding the absorption of light and the crystal structure's capacity to store energy caused by unbiased charge excitations [58]. The hypothetical dielectric function ε_2_(ω) contributed to the knowledge of the electronic bandgap, which is significant to the energy of the inter-band transitions close to the Fermi level. The ε_2_(ω) values of Sr_3_AsI_3_ occupied a significant section of the absorption zone. According to Fig. 12(c), the largest maxima of ε_2_(ω) for Sr_3_AsI_3_ were found at an optical position of 10.8, indicating that the energy of the absorption photon was close to 0.55 eV. The dielectric function's imaginary portion expands and swings toward the long wavelength area under applied strain, as shown in Fig. 12(d). These fictitious absorption peaks define the valence of the carriers to conduction band motion. The bandgap fluctuations and lattice constant variations lead the peaks to be offset. The imaginary peaks exhibit red-shift and blue-shift when compressive and tensile strains are applied (Fig. 12(d)). This condition suggests that tensile as well as compressive strain may result in a tunable absorption spectral region for the Sr_3_AsI_3_ compound under investigation. We also found that the fictitious dielectric fraction is zero for photon energies greater than 8 eV. The material's reduced optical absorption and improved optical transparency are revealed by the absence of ε_2_(ω) (above 8 eV).

**Fig. 13:**
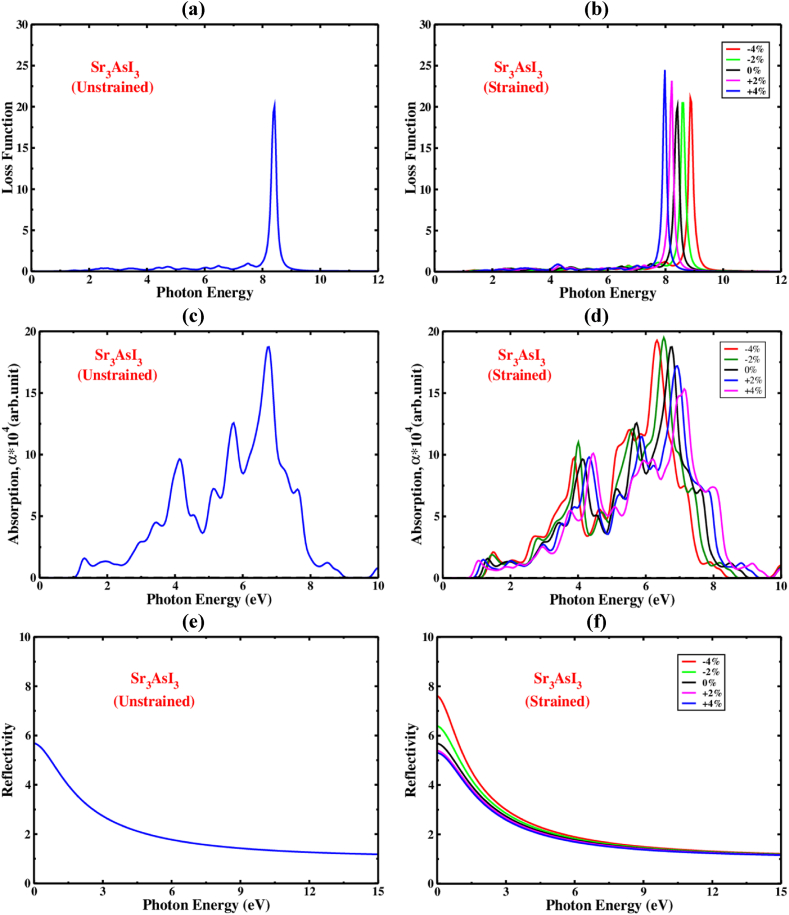
The loss function, absorption, and reflectivity as a photon energy function of (a,c,e) without strain, and (b,d,f) with strain Sr_3_AsI_3._

The amount of energy lost by electrons as they move through a dielectric substrate is known as the “electron loss function” [61]. It is represented by the symbol L(ω), and it also involves examining how a chemical responds to light exposure. The peak in Fig. 12(a)'s plot of L(ω) for Sr_3_AsI_3_ indicates that the energy loss is detected when the emitted photon's energy surpasses the material's bandgap. A formula allows us to see the loss function, L(ω) = j ($\frac{-1}{\varepsilon \left(\omega \right)}$). According to Fig. 13(a), the cubic Sr_3_AsI_3_ structure's L(ω) peaks emerged between 7 and 10 eV. The estimated electron loss function, L(ω), for Sr_3_AsI_3_ was quite significant at a value of 8.5 eV. Separately, the highest and lowest peaks of loss were discovered at 2.5 eV and 8.5 eV. According to the insignificant presence of L(ω) peaks below 2 eV, the Sr_3_AsI_3_ component would function as an efficient optical absorption layer in the area of the visible photon spectra and IR. It is revealed that the loss function of the applied strain corresponds to photon energies up to 10 eV of Sr_3_AsI_3_. Fig. 13(b) shows the predicted energy losses of Sr_3_AsI_3_ under various biaxial compressive and tensile strain conditions. It has been established that when the compressive strain for all constructions is raised, a sizable transformation to reduced photon energy is apparent via optical loss (redshift). Conversely, as the tensile strain is increased, the optical loss likewise exhibits a sizable shift to upper photon energies (blueshift). Overall, the loss function of Sr_3_AsI_3_ has a significant impact on performance, which is an important consideration when designing and optimizing these materials for specific applications. One essential property of Sr_3_AsI_3_ is its absorption coefficient, which expresses how strongly a compound absorbs light at various wavelengths. The Sr_3_AsI_3_ perovskite's absorption coefficient is influenced by a number of factors, including the material's purity, thickness, and crystal structure. The profile of the optical absorption coefficient for any configuration exhibits traits that are comparable to those of the imaginary component of the dielectric constant [58]. The visible portion of the electromagnetic spectrum, which normally includes the majority of the sun's radiation, typically has a higher absorption coefficient. As a measurement of photon energy without and with biaxial strain, the Sr_3_AsI_3_ perovskite material's absorption coefficient is depicted in Fig. 13(c) and (d), respectively. The absorption peak definitely exhibits a considerable blueshift with tensile strain, but it exhibits a significant redshift under compressive strain.

In the spectrum of visible light, the compressed structure has a stronger absorption capacity and the tensile structure has a lower absorption capacity than the unstrained one. The absorption coefficient of the Sr_3_AsI_3_ framework improves in the visible range with growing compressive strain, which is significant for solar cell implementation. On the contrary, the absorption coefficient of the Sr_3_AsI_3_ structure drops in the visible area as tensile strain is increased. Generally, the Sr_3_AsI_3_ perovskite absorption coefficient is a crucial factor to take into account while building photovoltaic systems based on this material. It might be possible to increase the effectiveness of these devices and make them more competitive with other types of solar cells by boosting the absorption coefficient. Reflectivity is the quantity of light that Sr_3_AsI_3_ perovskite reflects when exposed to electromagnetic radiation, such as visible light. The overall reflectivity of perovskite materials can differ significantly depending on factors like composition, crystal structure, and surface shape. Additionally, the Sr_3_AsI_3_ perovskite's reflectivity may be impacted by the wavelength and angle of incidence of the incident light. As a measurement of photon energy, the reflectivity of the Sr_3_AsI_3_ perovskite material is depicted in Fig. 13(e) and (f), respectively, with and without biaxial strain. Between 0 and 5 eV photon energy, the reflectance varies most noticeably. The maximum reflectivity is at 0 eV photon energy. The reflectivity of the Sr_3_AsI_3_ framework improves in the visible range with growing compressive strain, which is significant for solar cell implementation. On the contrary, the reflectivity of the Sr_3_AsI_3_ structure drops in the visible area as tensile strain is increased. The findings of this study on the optical properties of Sr_3_AsI_3_ match those of earlier papers pretty well [84]. Materials with bandgaps less than 3.1 eV perform better in visible light device applications [58]. Because of its unique optical properties, Sr_3_AsI_3_ perovskite is a promising candidate for use in a range of applications, such as solar cells, optoelectronics, and optical sensors.

## 4 Conclusions

The inorganic perovskite Sr_3_AsI_3_ has been studied in terms of its electronic, optical, and structural characteristics using first-principles DFT calculations. Our analysis of the strain-driven optical properties leads us to the conclusion that the Sr_3_AsI_3_ exhibits the peaks of absorption (blue-shift as well as red-shift) in the vicinity of the ultra-violate to visual spectrum at various induced strains. The direct bandgap of the perovskite Sr_3_AsI_3_ material was likewise determined to be 1.265 eV. The electronic bandgap value of Sr_3_AsI_3_ decreases to 1.212 eV in the presence of the SOC effect. The bandgap is observed to drop with growing induced compressive strain, including or excluding the SOC effect, but to rise with growing induced tensile strain. Furthermore, the peak of the dielectric function of Sr_3_AsI_3_ transfers to greater energy of the photon (blueshift) if the induced tensile strain is larger and shifts to smaller energy of the photon (redshift) if the induced compressive strain is greater. We believe that the results of our most recent examination will stimulate additional studies to create the well-known perovskite Sr_3_AsI_3_ for application in optoelectronic devices.

## Author contributions

Md. Ferdous Rahman, Avijit Ghosh, Md. Rasidul Islam: Conceived and designed the experiments; Performed the experiments; Analyzed and interpreted the data; Contributed reagents, materials, analysis tools or data; Wrote the paper.

Md. Shoriful islam, Abu Bakar Md. Ismail: Analyzed and interpreted the data; Contributed reagents, materials, analysis tools or data; Wrote the paper.

Mongi Amami, M. Khalid Hossain: Contributed reagents, materials, analysis tools or data; Wrote the paper.

## Data availability

Data will be made available on request.

## Declaration of competing interest

The authors declare that they have no known competing financial interests or personal relationships that could have appeared to influence the work reported in this paper.
